# Supplementary material for: Are carbon emissions trading and green financial instruments synergistic? -Comprehensive quantitative research based on content analysis
Source: PLoS One. 2024 Mar 7;19(3):e0298601. doi: 10.1371/journal.pone.0298601 (PMC10919601; doi:10.1371/journal.pone.0298601)
Supplement: S1 Data — (ZIP) [file pone.0298601.s001.zip › Data/地级市Z_F工作报告.zip!/╔╜╬≈/╤⌠╚¬╩╨/└┤╫╘╣1⁄2╓┌║┼í╛┬φ┐╦╩2╛▌═°í┐_2018.docx]

各位代表：

现在，我代表市人民政府向大会报告工作，请予审议，并请市政协委员和列席人员提出意见。

一、2017年工作回顾

过去的一年，我们坚持以习近平新时代中国特色社会主义思想为指引，坚决贯彻落实党的十九大精神和习近平总书记视察山西重要讲话精神，按照省委“一个指引、两手硬”思路和要求，在市委的坚强领导下，深入贯彻中央、省一系列会议精神，坚持稳中求进工作总基调，践行新发展理念，紧紧依靠全市广大干部群众，全力抓好稳增长、促改革、调结构、惠民生、防风险各项工作，经济发展实现由“疲”转“兴”，社会事业迈出新的步伐。

（一）经济增长步入合理区间。全市地区生产总值完成672亿元，同比增长6.3%，较近年来增速最低点回升了10.8个百分点。规模以上工业增加值完成217亿元，增长5.5%，规模以上工业企业主营业务收入增长21.3%，实现利税增长91%。九大产业重点项目加快推进，全社会固定资产投资完成245.7亿元，增长6.6%。社会消费品零售总额完成325亿元，增长6.1%。一般公共预算收入完成50亿元，增长21.1%，较上年回升了27.6个百分点。全市经济呈现稳中向好、好中向优态势，发展质量效益稳步提升。

（二）结构调整和动能转换成效明显。产业结构加快调整，三次产业比例为1.5∶47.7∶50.8，第三产业支撑作用进一步增强。工业结构持续优化，非煤产业对工业增长的贡献率达到84.8%。财政结构质量提升，税收收入同比增长38.5%，占一般公共预算收入的比重达到75.7%。煤炭供给侧结构性改革成效显著，压减落后产能210万吨，优质产能占比提升到64%。新登记各类市场主体13914户，同比增长12.6%。新培育“小升规”企业10家，高新技术企业达到42家，市级各类双创平台达到14家。经济结构发生积极变化，新旧动能正在接续转换，转型发展呈现出良好态势。

（三）重点领域改革取得新突破。开发区“一体两翼”格局正式确立，整合改制扩区调规工作扎实推进，“三化三制”改革正在落地，阳泉经济技术开发区扩区至80平方公里，平定省级经济技术开发区挂牌成立，盂县经济技术开发区完成组建等待批复。国企国资改革的政策体系已经建立，改革路径基本明确，厂办大集体改革、国有资本运营投资公司组建工作得到省政府充分肯定。行政托管工作取得突破，城区整建制托管郊区义井镇，矿区托管郊区平坦镇18个行政村。同时，旅游景区体制机制、投融资体制、国有林场等各项改革走在全省前列。“改革决不能落后”的要求在阳泉得到充分体现！

（四）“三农”工作焕发出新活力。农业供给侧结构性改革迈出坚实步伐，现代特色高效农业快速发展，设施蔬菜、小杂粮、食用菌、中药材等种植面积不断扩大。粮食总产量达到2.6亿公斤。脱贫攻坚再战再胜，贫困村退出47个、脱贫8978人，贫困发生率由建档立卡初期的4.1%下降到0.54%，为实施乡村振兴战略奠定了坚实的基础！

（五）城乡统筹基础更加巩固。阳大铁路、国道307、207绕城改线工程、中环快速路等一批覆盖城乡的基础设施项目进展顺利，公共停车场建设加快推进，道路交通秩序得到有效治理。以“六区六道”为重点的城乡环境集中整治行动迅速，措施有力，南大街、义平路综合整治成效显著。龙华口水库实现下闸蓄水。城乡人居环境进一步改善，城乡一体化发展水平再度提升，城镇化率达到67.56%，正向省级统筹城乡示范区目标迈进！

（六）生态环境质量得到明显改善。大气、水、土壤污染防治“三大战役”捷报频传，大气环境质量进一步好转，市区空气质量综合指数同比下降4.6%，PM2.5平均浓度同比下降3.2%，“大气十条”目标任务全部完成。地表水环境质量持续向好，省控以上优良断面比例达到省定要求。矿山生态环境恢复治理有序推进，工业固体废弃物利用率71.9%，超过全省平均水平。各类营造林完成10.02万亩，森林覆盖率达到28.5%。环保治理工作扭转了被动局面！侯卫东官场笔记

（七）民生社会事业取得长足进步。各级财政用于民生支出83.5亿元，占一般公共预算支出近八成。城镇新增就业2.53万人，转移农村劳动力1.16万人。城乡居民人均可支配收入分别增长6.4%、6.5%，增速跑赢GDP，农村居民收入增速持续快于城镇居民。义务教育均衡发展成果继续巩固，高中教育办学质量稳步提升，“一本两专”高等教育和较完整的职业教育体系基本形成。公立医院综合改革全面推进，县乡医疗卫生机构一体化改革取得阶段性成效。保障性安居工程建设任务超额完成，公租房分配率全省排名第三。公共文化服务体系不断完善，举办各类文化惠民活动2850场。群众体育迈入全国先进行列，竞技体育再创佳绩。安全生产形势持续好转，各类事故死亡人数下降16.13%，连续两年煤炭百万吨死亡率为零，成为全省唯一杜绝较大及以上事故的市。人民群众的获得感、幸福感和安全感更加充实！

（八）政府自身建设实现全面提升。严格落实政府系统全面从严治党主体责任，深入推进党风廉政建设和反腐败斗争。坚持依法行政，主动接受人大、政协监督，办理人大代表建议205件、政协提案336件。“放管服效”改革有序推进，取消下放行政职权事项107项，取消行政审批前置申请材料210项，政府部门保留行政许可事项全省最少，网上政务服务平台建设在全省领先。“阳泉随手拍”问政平台深得民心，“13710”督办系统高效运行。服务型政府建设取得新成效！

一年来，民族宗教、外事侨务、科普、档案、史志等工作取得新进展，妇女儿童、青少年、老龄、残疾人、红十字等事业有了新发展，国防动员、双拥、人民防空等各项工作呈现出新气象。

各位代表，困难中的转型来之不易，压力下的收获更为可贵。成绩的取得，根本上靠的是习近平新时代中国特色社会主义思想的科学指引和以习近平同志为核心的党中央的英明领导，靠的是省委、省政府和市委的坚强领导，靠的是市人大、市政协和社会各界的有效监督、大力支持，靠的是全市上下凝心聚力、团结奋斗！在此，我代表阳泉市人民政府，向全市人民，向各民主党派、工商联和无党派人士，向各位人大代表、政协委员，向驻地部队、公安民警和中央、省驻地单位，向所有关心支持阳泉发展的各界朋友，表示崇高的敬意和衷心的感谢！

在肯定成绩的同时，我们也清醒地认识到，我市发展仍面临不少困难和挑战。集中表现为：长期积累的结构性体制性素质性矛盾依然突出，“一煤独大”“一股独大”尚未得到根本性改变，传统产业后劲不足，新兴产业刚刚起步；民营经济弱小，创新动力、创新人才不足；营商环境不优，生态环境基础脆弱，环境治理压力巨大；城乡基础设施、公共服务及民生社会事业欠账较多，社会治理还有薄弱环节；政府职能转变还不到位，“放管服效”改革有待深入，干部思想观念、能力素质与新时代要求还有差距，等等。对此，我们一定本着对人民群众高度负责的态度，采取有力举措，切实加以解决。

二、2018年工作安排

2018年是全面贯彻党的十九大精神的开局之年，是改革开放40周年，是决胜全面建成小康社会、实施“十三五”规划承上启下的关键一年，也是深化供给侧结构性改革和转型综改试验区建设的重要一年。做好今年政府工作，要以习近平新时代中国特色社会主义思想为指引，全面贯彻党的十九大、十九届二中、三中全会、全国两会精神和省委省政府、市委的一系列安排部署，把党的领导贯穿于政府工作全过程，坚持稳中求进工作总基调，坚持以人民为中心的发展思想，深入践行新发展理念，全年工作以“123335”为基本思路，即始终围绕供改与综改相结合这条主线，牢牢把握两个根本，即以转型发展为根本出路，以高质量发展为根本要求，围绕省委提出的“示范区”“排头兵”“新高地”三大目标定位，做好构建多元支撑产业体系、建设清洁能源供应基地、打造对外开放桥头堡“三篇文章”，着力补齐民营经济弱小、创新动力不足、金融支撑不够“三个短板”，坚决打好防范化解重大风险、精准脱贫、污染防治“三场硬仗”，统筹抓好重点领域改革、优化营商环境、转型项目建设、乡村振兴战略实施、保障和改善民生“五项重点工作”，以坚定的决心、一流的标准、过硬的作风，大力推动质量变革、效率变革、动力变革，促进经济社会持续健康发展。

2018年全市经济社会发展的主要预期指标是：地区生产总值增长6.5%左右；规上工业增加值增长6%左右；固定资产投资增长9%；社会消费品零售总额增长7%；一般公共预算收入增长6.5%；城镇居民人均可支配收入增长6.5%，农村居民人均可支配收入增长6.5%以上；新增就业岗位2.2万个，城镇登记失业率控制在4.2%以内；居民消费价格涨幅控制在3%左右。

约束性指标是：万元地区生产总值能耗下降3.2%，万元地区生产总值二氧化碳排放量下降3.9%，万元地区生产总值用水量下降2.5%。环境质量改善指标和主要污染物总量减排指标，完成省下达年度目标任务。

围绕上述总体思路和目标任务，重点抓好以下八个方面具体工作：

（一）认真做好“三篇文章”，加快走出资源型经济转型发展新路

1、构建多元支撑的现代产业体系

按照新兴化、集群化、规模化的要求，立足优势延长链条，利用先进技术改造提升传统产业，全力培育壮大战略性新兴产业，加快构建多元发展、多极支撑的现代产业体系。

改造提升传统产业。提高煤炭产业产品附加值，原煤入选率保持在90%以上。着力培育优质产能，力争到2020年优质产能占比达到2/3以上。重点在“煤-电-铝-材”“煤-电-化”上下功夫，加快推进阳煤集团50万吨电解铝及轻质铝合金项目。推动阳煤平定乙二醇一期项目投产达效并抓紧筹备二期项目。电力产业要加快国际能源裕光煤电2×100万千瓦项目建设，推动总规模18万千瓦的4个风电项目开工，加快阳煤二矿桑掌煤层气发电项目建设，谋划启动光伏领跑者基地二期项目。装备制造业要重点提升煤矿机械、电气设备等专业装备制造能力，支持华越机械、华鑫电器、奥伦胶带等企业进行技术创新推动智能制造，建设面向全国的煤矿机械电气生产及服务基地。推动全市耐火材料产业结构调整，鼓励耐火骨干企业开展跨区域、跨所有制兼并重组，提高产业集中度。支持耐火企业上档升级，提高产品质量档次，发展高附加值、高技术含量、低能耗型耐火产品。

培育发展新兴产业。抓住国发42号文赋予阳泉智能物联网应用基地试点建设的有利时机，加快发展壮大新一代信息技术产业。编制全市智能物联网应用基地试点建设三年规划和2018年行动计划，建设智能物联网基础能力平台、产业交易平台和产业孵化基地，并在群众感知度高的领域，如环卫、交通、教育、安监等，开展应用示范。与百度开展更加紧密的合作，大力引进大数据、物联网、云计算等产业项目，推动爱康山西智慧能源综合服务平台、三和国投互联网呼叫中心二期等项目投运，加快云谷科技创新园建设。新材料产业以精深加工和提升附加值为方向，瞄准磁性材料、陶瓷、陶粒砂、石墨电极、活性氧化钙等领域，提高自主研发能力，培育壮大龙头企业。重点支持中兴环能纳米洋葱碳项目扩大产能，加快贝特瑞3万吨高端人造石墨等项目建设，支持特种陶粒砂、空心微珠等新材料企业做大做强。文旅康养产业要坚持高端策划，突出阳泉特色，融入全省黄河长城太行三大旅游板块战略，启动太行山一号旅游公路阳泉段建设。重点推进以药林寺、大汖温泉景区为依托的文旅康养项目建设。实现娘子关景区高起点、全要素快速升级。加快发展乡村旅游。年内全市旅游总收入增长20%左右。现代物流产业，依托我市区位和交通优势，做强做优煤炭物流，同时加快大型综合类、专业类物流园区建设，积极发展供应链物流、第三方物流、互联网+物流、冷链物流，推进中岚国际、顺康蒙牛、金亿通商贸等物流项目建设。

2、建设清洁能源供应基地

按照我省建设国家清洁能源基地的安排部署，结合阳泉实际，着力在能源供给、消费和体制机制方面取得突破，使我市成为华北地区重要的清洁能源供应基地。

大力调整能源供给结构。提高清洁能源比重，推动能源供给多元化。严格执行煤矿减量重组实施方案，完成阳煤三矿裕公井、燕龛煤矿、鸿泰煤矿3座矿井关闭任务，退出产能205万吨，积极引导60万吨以下矿井有序退出。加快推进盂县电厂-河北辛集外送电通道建设，提升区域电力外送能力。有序放开煤层气勘查开采准入，支持发展煤层气综合利用发电项目。大力引进先进技术，有序发展薄膜太阳能、风能、地热、生物质能等新能源。

不断优化能源消费结构。作为全省首批煤炭消费总量控制城市，坚决完成禁煤区燃料煤炭清零任务，大力发展清洁煤燃烧技术，实施优质型煤等清洁燃料置换，实现煤炭消费总量负增长。抓好工业领域节能，推进重点行业能效提升，在高耗能行业开展能效对标，加快现役煤电机组节能改造，新增产能主要耗能设备能效达到国内先进水平。加大对农村电网改造升级的支持力度。

加快完善能源体制机制。切实推进电力体制改革综合试点工作，继续组织企业参加电力直接交易，积极争取在阳泉经济技术开发区东区工业园开展增量配电业务，进一步降低企业用电成本。全面实现矿业权竞争性出让，实行更加严格的煤层气勘查区块退出机制和公开竞争出让制度。鼓励煤炭矿业权人和煤层气矿业权人合资合作，支持符合条件的企业和我市煤炭、煤层气开采企业开展合作。

3、打造对外开放桥头堡

充分发挥我市位于晋冀要冲的区位优势，以更加开放的视野和更加主动的姿态融入国家、省发展战略，在对外合作、交流、贸易上实现新提升，构建全方位开放新格局。

拓展合作交流。主动对接融入京津冀，加大与北京、天津、石家庄、太原等地区的合作，深度融入太原、石家庄都市圈，推进国际友城之间的经贸合作、文化交流，建设更加开放、更具活力、更有优势的外向型经济体系。积极参与承办国际国内各类专业化论坛、大型赛事、文旅活动，扩大城市知名度与美誉度。支持鼓励外资企业的发展。积极培育外贸主体，扩大外贸规模，优化出口产品结构，努力做大做强新材料、日用陶瓷等出口品牌。

搭建开放平台。跟随国家“一带一路”倡议，抓住国家环保政策和供给侧结构性改革机遇，加快推动山西（阳泉）国际陆港建设，融合公路、铁路、海运多种运输方式，发展钟摆式多式联运物流链。通过智能物流云平台，基本形成联结晋陕蒙与环渤海港口、长江沿江码头的现代物流网络。启动中心商务区、综合保税区建设，努力建成联结我国东西部经济发展的现代化、智能化、国际化物流网络。把我市建设成为联结“一带一路”的开放大通道。

加大引资力度。强化招商引资激励机制，今年市政府安排500万元专项资金，对超额完成任务指标或引进重大项目的县区予以奖励。围绕我市产业转型主攻方向，突出以商招商，扩大委托招商，积极探索开展网上招商和以资招商。深化乡情招商，继续开展“晋商晋才回乡创业创新、泉心泉意共促阳泉发展”招商活动。强化跟踪服务，推动招商引资项目尽快落地，提高招商项目履约率和资金到位率。年内全市引进5亿元以上项目25个，完成招商引资签约任务518亿元，到位资金151亿元。

各位代表！机遇稍纵即逝，转型迫在眉睫。我们要按照省委提出的“示范区”“排头兵”“新高地”三大目标定位，找准突破口，攻坚克难，奋力拼搏，打出转型组合拳，努力闯出一条结构优、质量高、效益好的转型发展新路！

（二）着力补齐“三个短板”，进一步提高发展质量

现代化的经济体系必须有现代化的产业体系作为支撑，产业体系的现代化不仅要多元发展、多极支撑，还需要实体经济、科技创新、现代金融、人力资源协同发展，这就要求我们必须补齐发展短板，提高发展系统性。

1、补齐民营经济弱小的短板，提高经济发展内在活力

民营经济在稳定增长、促进创新、增加就业、改善民生等方面具有重要作用。我们要努力创造更加宽松的环境，激发各类市场主体活力，推动民营经济蓬勃健康发展。

创造良好的政策和法治环境。认真落实支持非公经济发展的各项政策举措，按照非禁即入的原则，全面实施市场准入负面清单制度，保障权利平等、机会平等、规则平等。加强企业家队伍建设，营造健康成长环境，构建“亲”“清”政商关系，让民营企业家放心经营、放手发展。完善产权保护措施，支持司法机关甄别纠正一批社会反映强烈的产权纠纷案件。制定出台支持民营经济发展的金融政策，深化政银企保合作，构建财政-银行-担保三位一体的政策体系，努力扩大银行对小微企业贷款投放规模。积极推动政府与社会资本合作，遴选一批有较好盈利预期、适合民间资本进入的优质项目，鼓励民间资本组建联合体参与建设。推进干部入企服务常态化，帮助企业解决发展难题。

积极扶持各类市场主体成长。按照“个转企、小升规、规改股、股上市”的路径，大力实施中小微企业成长工程。创新中小微企业融资模式，筛选、帮助一批规范化股份制改造的企业实现上市挂牌直接融资。积极推动中兴环能等公司在新三板挂牌。鼓励民营企业发行债券。建立“小升规”帮扶工作机制。全年新培育小微企业1500户以上，新增规上企业7户以上。

2、补齐创新动力不足的短板，增强经济发展内在动力

创新是引领发展的第一动力。我们要以创建国家级创新型城市为引领，加快建立企业为主体、市场为导向、产学研深度融合的创新体系，促进新旧动能加快转换。

持续优化创新创业生态。加大科技企业孵化器、双创示范基地、众创空间培育力度，探索建立龙头示范、优势互补、共同发展的孵化联盟体系。积极开展对创新创业项目的“一条龙”“一帮一”服务。以阳泉高新技术创业园国家级孵化器为龙头，提升省级以上双创平台运营服务能力。抓好山西工程技术学院大学生创业孵化基地建设，推进百度创新中心、大唐阳泉369云工厂等项目，支持矿区依托阳煤集团建立专业性众创空间。举办好“五小”创新竞赛、青年创新创业大赛等活动。

切实加快创新成果转化。强化企业创新主体地位，引导企业加大研发投入，支持企业与高校、科研院所共建各类研发平台，鼓励企业设立或并购研发机构。加快实施一批重大专项及重点科技项目。积极培育知识产权优势企业和专利试点企业，完善科技成果转化和知识产权交易平台，建立科技成果转化引导基金，打通科技创新最后一公里。

不断夯实创新人才支撑。全面落实我省出台的以增加知识价值为导向的十四项配套政策，在全市医疗机构、科研院所落实用人、职称评审、工资分配三项自主权。加大对知识密集、人才集中事业单位绩效工资总量倾斜力度。研究出台支持大学毕业生回乡创业就业的政策措施。建立与各类智库、决策咨询机构的常态化合作机制，引进高端科技创新人才、高级经营管理人才和高技能工匠人才。大力支持院士工作站、技能大师创新工作室等平台建设，加大本地专业技术人才培养力度。

3、补齐金融支撑不够的短板，提高服务实体经济能力

金融是实体经济的血脉，任何时候都不能脱实向虚。我们要把握回归本源、优化结构、强化监管、市场导向的原则，推动金融业为高质量发展提供有力支撑。

营造良好的金融生态。推进银行业机构健康发展，充分发挥融资主渠道作用，加大信贷资金投放力度。支持金融机构围绕实体经济创新金融产品和服务，开展“文创贷”“商易贷”等业务，重点支持新兴产业发展和转型项目建设。创新政策性融资担保机构经营模式，利用专项建设基金、应急周转保障资金等渠道降低企业融资成本。

加快地方金融机构改革。支持市商业银行完善法人治理结构和治理机制，优化股权结构，尽快更名为阳泉银行。加快推进平定、郊区农村信用社改制农村商业银行工作。规范盂县汇民、平定昌都等村镇银行业务经营，加强股东治理和违规整顿。支持企业财务公司发挥业务优势，提升服务能力。支持金融机构开展普惠金融业务，推广普惠金融产品。

有效发挥产业基金作用。今年市政府将设立1亿元的转型产业引导基金，主动对接省级产业投资基金，充分发挥财政资金的引领撬动作用，探索与其他风投资金进行合作，有效改善企业资本供给。引导资金投资方向，推动一批极具创新能力、市场前景好的初创期企业快速成长。

各位代表！发展的短板不会自动补齐。我们要坚持问题导向，盯住痛点下功夫，抓住短板齐发力，为我市协调发展、可持续发展厚植土壤，创造有利条件！

（三）坚决打好“三场硬仗”，进一步夯实发展基础

1、坚决打好防范化解重大风险攻坚战

下大力防范化解金融风险。要强化属地风险处置责任，建立风险监测预警和早期干预机制，加强对小额贷款公司、融资担保机构的监管，强化对个体网络借贷和网络小额贷款的整治，严防严打非法集资、传销等涉众型经济犯罪，做到金融监管全覆盖。今年要特别对政府债务加强管理，开前门、堵后门，双管齐下解决好债务问题。开前门就是要合理安排新增债务规模，合法合规举债融资；堵后门就是要严格落实地方政府债务限额管理和预算管理制度，防止盲目过度超前、不考虑偿还能力举债。

加强和创新社会治理。提高社会治理社会化、法治化、智能化、专业化水平，打造共建共治共享的社会治理格局。推动社会治理重心向基层下移，实现政府治理和社会调节、居民自治良性互动。提高预警、预判、预防和应急处置能力，严防重大公共安全事件发生。加强预防和化解社会矛盾机制建设，及时妥善处理重点领域矛盾纠纷。加强网络安全防护工作。深化平安阳泉建设，狠抓社会治安综合治理，推进扫黑除恶专项斗争，打击黄赌毒、盗抢骗等各类违法犯罪，推进综治视联网、“雪亮工程”建设。

2、坚决打好精准脱贫攻坚战

突出精准脱贫施策重点。加快推进“一村一品一主体”建设，实施特色产业扶贫，夯实稳定脱贫基础。加强基础设施建设和环境综合整治，不断提升贫困村村容村貌。强化扶贫工作队伍日常管理，做到包村领导包到位、帮扶单位帮到位、党员结对做到位，提升帮扶精准度。

办好精准脱贫保障实事。加强教育扶贫，落实各学龄段补助政策，开展优势教学资源校际交流帮扶。加强健康扶贫，落实“三保险三救助”“双签约”政策，完善先诊疗、后付费一站式结算平台，简化大病、慢性病认定程序。加强就业培训，抓好转移就业，支持鼓励创业。加强兜底保障，提高农村低保补助标准。

强化精准脱贫推进机制。坚持市县抓落实、党政一把手负总责。继续加大财政投入和涉农资金整合力度。积极开展公益活动和民营企业助脱贫活动，鼓励社会力量参与扶贫事业。加强宣传引导，形成浓厚的脱贫攻坚决战氛围。激发内生动力，推进“领帮带”模式在贫困村全覆盖，推广孝亲敬老补助政策。强化监督考核，深入开展扶贫领域作风建设年活动，严肃查处扶贫领域的不正之风和腐败问题。今年完成32个贫困村退出、3325人脱贫，在全省率先实现整体脱贫。

3、坚决打好污染防治攻坚战

大力推进环境污染综合整治。狠抓大气污染治理。继续抓好“控煤、治污、管车、降尘”等举措，坚决打赢蓝天保卫战。加大散煤整治力度，积极稳妥推进冬季清洁取暖工程，保留的燃煤锅炉全部实施特别排放限值改造。巩固“散乱污”企业取缔成果，杜绝异地转移和死灰复燃。严格落实货运车辆禁限行措施。加强建筑工地扬尘管控，加强道路清扫和洒水抑尘，继续推进储煤场、物料堆场全封闭改造。认真做好重污染天气应对工作，严格执行错峰生产和应急减排规定。狠抓水污染治理。全面落实河长制责任。推进煤矿矿井水提标改造。加快工业集聚区、建制镇污水处理设施建设。完成蒙村河、洪城河、桃河下游城区段3条黑臭水体治理。建成投用市污泥处理处置中心项目。完善规模化畜禽养殖场粪污治理设施。继续推进排污许可制度改革。加强娘子关水源地保护工作。狠抓土壤污染治理。推进土壤污染状况详查，加强土壤环境监测监管能力建设，实施农用地土壤环境分类管理。加强工业废物处理处置。狠抓环境问题整改。认真做好中央、省委省政府环保督察和环保部督查巡查问题整改，采取建账、对账、查账、销账、交账“五账工作法”，推动铁腕治污常态化，始终保持环保严打高压态势。加强生态环境修复治理。加快推进采煤沉陷区治理搬迁安置未竣工项目建设。加强矿山生态恢复治理，争取用2-3年时间基本治理矸石山污染问题。加强生态保护修复，完成各类营造林6.5万亩、水土流失治理13.95万亩。

改革完善生态环境管理体制。实行最严格的生态环境保护制度，构建政府为主导、企业为主体、社会组织和公众共同参与的环境治理体系。推进生态文明建设目标考核评价工作，建立多元化生态补偿机制，统筹推进环保信用评价、生态环境损害赔偿制度改革、生态保护红线划定等工作。落实生态环保责任制，严格执行领导干部离任审计和责任追究规定。

各位代表！三大攻坚战打得好不好，直接关系到全面建成小康社会质量高不高。我们必须牢固树立底线思维，拿出过硬措施，攻坚克难，为满足人民日益增长的美好生活需要而不懈奋斗！

（四）抓好重点领域改革，为深度转型添动力

深化开发区改革创新。深化体制机制改革。持续深入推进“三化三制”改革。落实好税收优惠政策。切实向开发区授权放权，确保放权充分，授权到位。提升平台建设水平。做好开发区总体规划、控制性详规和规划环评报告编制。加快新扩区和起步区建设。加快完善基础设施和公共服务平台建设，做好“七通一平”熟地供应，变“项目等地”为“地等项目”。推进转型项目建设。把招商引资和开发区建设有机统一起来，引进一批重大转型项目。今年，阳泉经济技术开发区新建5个投资超10亿元的转型项目，平定、盂县开发区各新建2个投资超5亿元的转型项目。

深化国企国资改革。紧紧抓住当前难得的窗口期，按照“1+7”政策体系和“五个一批”路径，加快推进国企国资改革。混合所有制改革发展壮大一批。加快水泵阀门退城进园、搬迁改制步伐，蔬菜公司新建农产品批发市场年内投运，完成市自来水公司及污水处理厂特许经营改革，探索市煤气公司改革方案，推动晋东化工厂搬迁。国有股退出放开搞活一批。对资产规模较大企业，通过引进社会资本和企业职工参股等方式实施改革。对资产规模较小企业，鼓励企业职工入股买断。完成市药材公司与国药集团山西公司合作组建批零公司，推进市食品总厂改革。“僵尸企业”清理退出一批。信建资产公司所属5户企业和建工集团所属机械化施工分公司实施歇业关闭，电石有限公司实施破产关闭。深化完善改革一批。对专业化职能企业，完善优化法人治理结构和资本结构。整合重组具有较强行业特殊性企业。精简整合企业改制留守处。加快内部改革一批。对物资集团、建工集团、信达投资管理公司、粮油公司和交通集团等集团类公司，强化内部管理，优化资本结构，推进下属二级企业改革，提高集团运营效率。同时，利用长期停产企业现有的闲置厂房、土地和设备等资产，通过招商引资，引入集生产、消费、娱乐于一体的新产业新业态，实现闲置国有资产有效利用。积极推进“两办”改革，以阳煤集团“三供一业”分离移交重组工作为重点，按照成熟一批分离一批的原则，稳妥推进国有企业分离办社会职能工作；年内完成厂办大集体改革任务。

深化政府机构改革。按照中央和省委关于政府机构改革的各项安排部署，加强和完善政府经济调节、市场监管、社会管理、公共服务、生态环境保护职能，优化机构设置和职能配置，形成职责明确、依法行政的政府治理体系。

同时，统筹抓好电力体制、财税体制、综合行政执法体制、旅游景区体制机制、医药卫生体制、行政区划调整等各项改革任务。

各位代表！当前发展和改革高度融合，发展前进一步，就需要改革前进一步。我们要按照省委省政府的要求，擦亮综改试验区“金字招牌”，用好先行先试“尚方宝剑”，打破体制机制障碍，突破制约发展瓶颈，推动我市重点领域改革挺进全省第一方阵！

（五）全力优化营商环境，进一步改善发展条件

全面推行企业投资项目承诺制。认真总结开发区试点经验，进一步简化程序、优化流程，不断完善具体操作办法，按照政府靠前服务、企业信用承诺的方式，推行清单告知、平台办理、流程再造、多图联审、收费管理“五统一”，变先批后建为先建后验，进一步确立企业投资主体地位，释放企业投资兴业活力。

继续深化行政审批制度改革。进一步取消行政审批事项，凡是国内其他设区市不再审批的事项，原则上我市也一律不再审批；凡是其他地方在规定时限内能做到的事情，我们也要做到。承接落实好省取消下放行政审批事项。严格执行权力清单、责任清单、负面清单管理制度。加快推进证照分离改革。继续深化“两集中、两到位”工作，整合各类公共资源交易平台，实现大厅之外无审批、平台之外无交易。

全面推进投资项目并联审批。进一步优化建设项目并联审批方案，建立高效运转的工作机制。做好投资项目在线审批监管平台与政务服务网上平台的对接工作，将投资项目的全过程审批监管事项纳入市级政务服务平台，推行并联审批模式。

大力推进“互联网+政务服务”。完成市政务服务中心搬迁工作。市县两级全面推行网上审批改革，全面运行市级政务服务平台，开通县区级平台并与市级平台实现对接，努力做到能网上办理的事项全部网上办理，力争今年在全省率先实现网上审批，让企业、群众少跑路直到不跑路。

加强事中事后监管。改变重审批轻监管的行政管理方式，把更多行政资源从事前审批转到加强事中事后监管上来。全面推行“双随机、一公开”，加快推进政府监管信息共享。加强信用体系建设，加大信息公开力度，建立完善守信联合激励和失信联合惩戒机制，促进市场主体依法诚信经营，营造诚信社会环境。

各位代表！优化营商环境就是解放生产力、提升竞争力。在转型发展关键时期，我们要进一步对表对标、自我革新，全力营造“六最”营商环境，让政府的主动服务、优质服务使群众舒心、企业顺心！

（六）抓好转型项目建设，为经济发展提供有力支撑

认真谋划储备转型项目。突出成长性原则、集聚性效应、产业链方向，积极谋划储备一批转型项目，建立市级项目储备库和行业项目储备子库，实行分级储备、动态管理。全市储备项目计划总投资达到492亿元以上。

全力加快转型项目建设。开展项目集中开工月活动，迅速掀起开工热潮，到4月底，续建项目全部复工，新建项目开工30%以上；到6月底，新建项目开工率达到80%以上。发挥重点工程示范带动作用，继续推行领导包保、挂牌督办制度，开展“进工地、到一线、解难题”活动，严厉打击恶意阻工扰工行为，推进在建重点工程无障碍施工。加快项目建设投产，合并简化验收程序，推进竣工验收事项一站式完成，同步完成配套设施建设，推进项目早日达产达效。

强化项目要素保障。扎实推进土地占补平衡、压覆矿产、盘活闲置低效利用土地等各项工作，最大程度保障转型项目用地需求。积极争取上级各类资金和银行信贷资金支持，培育发展创业投资、风险投资、股权投资，广泛吸引社会资本，最大限度保障转型项目资金需求。加大高耗能、高污染项目节能减排力度，腾出环境容量，重点保障转型项目需求。同时，主动靠前服务，保障项目建设和生产运营过程中的用电、用水、用气等需求。

完善推进工作机制。严格落实责任，明确各县区政府和市直有关部门主要领导为推进项目建设的第一责任人，按照区域管理、部门职责，建立项目和拟办事项台账，明确具体事项办结时间。强化协调调度，每月召开一次投资项目调度会，每双月召开一次现场观摩会，协调解决突出问题。对各县区项目建设工作情况逐月进行排名、公示，排名末位的，对其主要领导进行约谈。加强督查考核，对推进缓慢的项目开展专项督查，将需要协调和解决的问题纳入“13710”督办系统进行跟踪督办、限期办结。

各位代表！项目兴，则经济兴；产业强，则阳泉强。我们要按照转型项目建设年工作方案，加快推进建设一批基础性、战略性、带动性强的优质项目，为我市转型发展夯实基础、积蓄力量！

（七）大力实施乡村振兴战略，加快推进城乡统筹发展

突出规划先导作用。以城乡统筹示范区建设规划为统揽，坚持乡村振兴与城乡融合一体设计，全面启动统筹城乡一体化总体规划和5个专项规划编制工作。规划要体现近中远期相结合，为全市实施乡村振兴战略、加快城乡统筹示范区建设指明方向与路径。

加快构建现代农业产业体系、生产体系和经营体系。大力发展特色高效农业，壮大杂粮、畜禽养殖、干鲜果、设施蔬菜、中药材、物流业六大产业。发展休闲农业，打造田园综合体，推进农林文旅康融合发展。实施区域品牌提升行动，抓好“三品一标”认证工作，做强半沟红薯、富硒小米、阳泉压饼等品牌。扎实推进农田水利基本建设，完成高标准农田建设9100亩。发展有机旱作农业，设立10个集成旱作农业技术推广示范点。提高现代农业生产机械装备水平。积极培育新型农业经营主体，以龙头企业为核心，以农民合作社为纽带，以专业大户和家庭农场为基础，构建股份制、合作制等紧密型利益联结机制。发展“互联网+农业”，推动农村电子商务发展。

推进美丽宜居乡村建设。深入开展美丽宜居示范村三级联创，持续推进农村人居环境改善四大工程，重点抓好农村垃圾污水治理、厕所革命、“四好农村路”建设等工作。加强传统村落保护和农村风貌管控。继续抓好郊区西南舁乡美丽宜居示范村集中连片建设，年内创建3个省级、10个市级美丽宜居示范村。

提升乡村文明程度。推进文明村镇、星级文明户、文明家庭创建工作，引导农民移风易俗，自觉抵制大操大办、厚葬薄养、人情攀比、天价彩礼等陈规陋习，培育文明乡风。强化“六议两公开”等民主治村工作程序，培育服务性、公益性、互助性农村社会组织，推进农村法治文化建设，发挥道德力量的导向作用，健全法治、德治和自治相结合的乡村治理体系。

全面深化农村改革。深化农村土地制度改革，在土地确权颁证的基础上，落实所有权、承包权、经营权“三权分置”办法，促进土地有序流转。推进农村集体产权制度改革，全面开展清产核资、规范管理和集体成员身份确认等工作，重点抓好郊区省级农村集体产权制度改革试点。有序推进集体经营性资产股份合作制改革，探索农村集体经济新的实现形式和运行机制。继续深化林权、水权制度改革。

进一步完善城乡基础设施。积极推进阳大铁路、国道307、207绕城改线工程、中环快速路等一批重大基础设施项目建设，鼓励支持社会资本依法依规参与建设。扎实推进生态修复、城市修补，加快桃河湿地公园建设。不断完善公共服务设施，完成市规划展览馆、市博物馆建设和布展，积极推进公共停车场、市中心医院等项目建设。支持娘子关镇申报国家特色小镇，支持盂县西烟、郊区河底等6个重点镇建设省级特色小城镇，提高城镇对人口和产业的承载力。

大力开展“五城联创”。创建省级文明城市。全面实施行政效能创优、市容整治优化、文明品牌创建等工程，进一步提升城市文明程度，切实增强全体市民的凝聚力和向心力。创建国家卫生城市。深入开展城乡环境综合整治行动，积极推进建筑垃圾、餐厨垃圾和有机废物、医疗废物等综合处置利用项目建设，创新引入以智慧环卫物联网管理为核心的信息化平台，努力创造优美整洁的城市环境，进一步提升城市品位。创建国家智慧城市。以智能物联网应用基地试点建设为契机，不断拓展应用领域，积极推进全市政务数据共享平台、公共信用信息共享平台、公共安全视频监控资源共享平台等项目建设，不断提升城市管理的智能化水平。创建国家创新型城市。加强创新投入，推进科技管理体制改革，加快实施科技政策创新、企业创新主体建设等重大创新工程，进一步提升城市的自主创新能力和科技发展水平。创建全国双拥模范城。扎实开展各项双拥活动，健全社会化双拥长效机制，认真做好转业退役军人就业安置工作，落实好军人军属优待政策，大力推进军地合力、军民同心，努力开创双拥工作新局面。

各位代表！实施乡村振兴战略是新时代做好“三农”工作的总抓手。我们要通过开展“五城联创”，以城带乡、城乡融合，推动乡村产业振兴、人才振兴、文化振兴、生态振兴、组织振兴，加快建设省级城乡统筹示范区，努力实现农村美、农业强、农民富的目标，在全省率先走出一条城乡一体、绿色发展的道路！

（八）坚持以人民为中心的发展理念，持续保障和改善民生

稳定扩大就业。完善就业创业政策，统筹做好高校毕业生、去产能职工、农村转移劳动力、“4050”人员、返乡农民工、退役军人等重点群体就业工作。加大就业专项资金支持、失业保险基金帮扶和企业主体责任落实力度。组织以市场需求为导向的就业培训，提升劳动者素质和就业能力。

提高社会保障水平。更好地执行城镇职工、城乡居民基本养老保险制度，落实被征地农民参加基本养老保险政策，建立社会保障待遇正常调整机制。继续提高低保、五保标准。完善社会救助体系，加大医疗救助和临时救助力度，探索支出型贫困救助模式。

发展公平而有质量的教育。推进县域内城乡义务教育一体化发展，教育投入继续向困难地区和薄弱环节倾斜，在办好优质均衡教育上先行一步。按规划完成消除大班额任务，有效解决中小学生课外负担重、择校热等问题。扩大普惠性学前教育资源供给。做好高中阶段学校考试招生制度改革试点工作，全面启动以高考改革为核心的高中教育改革。建成并启用阳泉职业技术学院新校区。

提升全民健康水平。继续推动医疗、医保、医药联动，努力解决看病难、看病贵问题。深化公立医院综合改革，完善政府办医体制。持续推进县乡医疗卫生机构一体化改革。加强基层医疗卫生服务机构标准化规范化建设。推进医保支付方式改革。推进和规范家庭医生签约服务。加强食品药品监管。以健康阳泉为目标，努力为人民提供全方位、全生命周期的健康服务。

改善住房保障能力。加强保障性住房建设管理，实现保障房体系覆盖所有困难群体。继续推进城中村改造、非成片棚户区改造。做好房地产去库存工作，确保供求关系稳定。积极推进住房制度改革，加快建立多主体供应、多渠道保障、租购并举的住房制度。

提升公共文化体育服务水平。提高公共文化服务普惠度，推进县级文化馆、图书馆总分馆制等改革。加快文化产业发展步伐，实施文化市场主体培育工程。围绕“不忘初心、牢记使命”主题教育，创作和打造一批文艺精品。加大文物和非遗保护力度。做好多功能文化综合体项目前期工作。完成第二届全国青年运动会射击射箭比赛场馆建设，做好第十五届全省运动会参赛及比赛项目承办工作。广泛开展全民健身活动，多渠道增加全民健身场所和设施。

下大力抓好安全生产。严格落实党政领导责任、部门监管责任和企业主体责任。加大执法检查力度，严厉打击安全生产非法违法行为，重拳打击私挖滥采和盗采国家矿产资源行为。继续深化煤矿、道路交通、危化品、建筑施工、城镇燃气、消防等重点行业领域专项整治，规范建立问题整改、挂牌督办、追责问责、联合惩戒四个清单。深入推进遏制重特大事故试点城市工作，构建完善风险分级管控和隐患排查治理双重预防工作机制。健全公共安全体系，提升防灾减灾救灾能力。

各位代表！人民对美好生活的向往就是我们的奋斗目标，我们所做的一切工作，都是为了人民。今年，市政府将在做好全面工作的基础上，重点办好10件民生实事。其中，省定民生实事5件：实施1460名残疾预防重点干预和残疾儿童抢救性康复项目，继续为怀孕妇女提供免费产前筛查与诊断服务，新建30个农村老年人日间照料中心，免费送戏下乡550场，实施全民技能提升工程。市定民生实事5件：提高城乡低保和特困供养标准，提高城乡居民基础养老金标准，规划建设6个城市公共停车场1500个停车位，实施10万平方米老旧小区综合整治工程，实施20个矸石山生态环境恢复治理示范工程。

同时，我们要坚决服务服从国防和军队调整改革大局，积极支持国防和军队建设，切实加强民兵和预备役部队建设，不断提升国防动员能力，提高国防教育质量，推动军民融合深度发展，创造军政、军民团结良好局面。

各位代表！我们的事业是全面发展的事业，是全市人民同心共建的事业。我们要支持工会、共青团、妇联等群团组织开展工作，加强气象、地震、人防、科普、政策咨询、经济普查、档案等工作，做好民族宗教、外事侨务、对台、对口援疆等各项工作，发展妇女儿童、老龄、慈善等各项事业，为我市改革发展凝聚强大合力！

三、努力建设人民满意的服务型政府

各位代表！进入新时代，开启新征程，政府工作必须要有新气象新作为。我们要以政治建设为统领，全面加强政府自身建设，深入推进政府职能转变，努力建设人民满意的服务型政府。

加强政治建设。自觉增强“四个意识”，坚定“四个自信”，坚决维护以习近平同志为核心的党中央权威和集中统一领导，始终在政治立场、政治方向、政治原则、政治道路上同党中央保持高度一致，不折不扣落实中央、省委和市委重大决策部署，把党的领导贯穿于政府工作全过程。深入推进“两学一做”学习教育常态化制度化，扎实开展“不忘初心、牢记使命”主题教育。

坚持依法行政。拥护宪法、尊崇宪法、维护宪法、学习宪法、宣传宪法，把政府活动全面纳入法治轨道。依法接受人大及其常委会的监督，自觉接受人民政协的民主监督，主动接受社会和舆论监督，认真听取各民主党派、工商联、无党派人士和各人民团体的意见建议。加强审计监督。严格规范公正文明执法，全面落实行政执法责任制。加快构建职能科学、权责法定、执法严明、公开公正、廉洁高效、守法诚信的法治政府。

提高政府效能。提升政府工作人员适应新时代、实现新目标、落实新部署的履职能力，着力解决不会为的问题，增强善作为的本领。全面推进政务公开，做好政务信息系统整合共享省级试点工作。强化督查考核，充分发挥“13710”督办系统的作用，促进各项工作部署落实到位。持续加大“阳泉随手拍”问政平台反映问题的解决力度，发挥好政府与群众之间的连心桥作用。以真抓的实劲、敢抓的狠劲、善抓的巧劲、常抓的韧劲，一步一个脚印，干出实打实的业绩，以实际行动赢得人民的信任和支持。

抓好作风建设。严格落实全面从严治党要求，深入推进政府系统党风廉政建设和反腐败斗争。坚决贯彻落实中央八项规定，驰而不息纠正“四风”，重点整治形式主义、官僚主义新表现。完善干部考核评价机制和激励约束、容错纠错机制，旗帜鲜明地为积极干事者撑腰鼓劲，大力整治和严肃查处不作为、慢作为、乱作为等问题，确保政府工作人员廉洁修身，勤勉尽责，清清白白做人，干干净净做事。

各位代表！阳泉山川秀美、矿产丰饶、红色传承、生态宜居，是一座名副其实的山水城、能源城、英雄城、幸福城。我们坚信，在全面建成小康社会的伟大进程中，勤劳勇敢、善良朴实的阳泉人民一定能够秉承优良传统，弘扬奋斗精神，继续创造出无愧于时代、无愧于历史的崭新业绩！

各位代表！新时代是奋斗者的时代。让我们更加紧密地团结在以习近平同志为核心的党中央周围，全面贯彻落实党的十九大精神，在省委、省政府和市委的坚强领导下，锐意进取、扎实工作，以转型发展、高质量发展的新业绩，谱写中国特色社会主义建设的阳泉新篇章！
